# *Arsenophonus nasoniae* and *Rickettsiae* Infection of *Ixodes ricinus* Due to Parasitic Wasp *Ixodiphagus hookeri*

**DOI:** 10.1371/journal.pone.0149950

**Published:** 2016-02-22

**Authors:** Monika Bohacsova, Oleg Mediannikov, Maria Kazimirova, Didier Raoult, Zuzana Sekeyova

**Affiliations:** 1 Department of Rickettsiology, Institute of Virology, Biomedical Research Center, Slovak Academy of Sciences, Bratislava, Slovakia; 2 Unite de Recherche sur les Maladies Infectieuses et Tropicales Emergentes (URMITE) IRD 198, CNRS 7278, INSERM 1095, Institut Hospitalo-Universitaire (IHU) Mediterranee-Infection, Aix-Marseille University, Marseille, France; 3 Department of Medical Zoology, Institute of Zoology, Slovak Academy of Sciences, Bratislava, Slovakia; University of Maryland, College Park, UNITED STATES

## Abstract

*Arsenophonus nasoniae*, a male-killing endosymbiont of chalcid wasps, was recently detected in several hard tick species. Following the hypothesis that its presence in ticks may not be linked to the direct occurrence of bacteria in tick's organs, we identified *A*. *nasoniae* in wasps emerging from parasitised nymphs. We confirmed that 28.1% of *Ixodiphagus hookeri* wasps parasitizing *Ixodes ricinus* ticks were infected by *A*. *nasoniae*. Moreover, in examined *I*. *ricinus* nymphs, *A*. *nasoniae* was detected only in those, which were parasitized by the wasp. However, in part of the adult wasps as well as in some ticks that contained wasp's DNA, we did not confirm *A*. *nasoniae*. We also found, that in spite of reported male-killing, some newly emerged adult wasp males were also infected by *A*. *nasoniae*. Additionally, we amplified the DNA of *Rickettsia helvetica* and *Rickettsia monacensis* (known to be *Ixodes ricinus*-associated bacteria) in adult parasitoid wasps. This may be related either with the digested bacterial DNA in wasp body lumen or with a role of wasps in circulation of rickettsiae among tick vectors.

## Introduction

*Arsenophonus nasoniae* is a rod-like Gammaproteobacterium with a large genome and a substantial metabolic capability [1;2]. In laboratory conditions it is cultivable in supplemented cell-free media [3]. *Arsenophonus nasoniae* harbours unusual combination of pathogenic and symbiotic features. It was originally described as an endosymbiont of the wasp *Nasonia vitripennis*, yet induces the so-called male killing phenotype—production of female biased secondary sex ratio associated with the death of 80% of male offspring [4;5]. Unlike other intracellular bacteria, *A*. *nasoniae* was found only within the somatic tissue and interstitial fluid surrounding the germ cells. These bacteria are ingested by feeding wasp larvae and invade its body through the gut, thus capable to re-infect the reproductive and other tissues of the parasitoid [6]. *Arsenophonus nasoniae* was described in several parasitoid wasp species which belong to the Chalcidoidea superfamily, e.g.: *Nasonia longicornis*, *Spalangia endius*, *Spalangia cameronii*, *Eupelmus vesiculari*, *Pachycrepoideus vindemmiae* and *Muscidifurax raptor* [7;8].

The chalcid wasp, *I*. *hookeri*, was reported in Central Europe for the first time by Bouček and Černý [9]. However, it is distributed worldwide and parasitizes a broad range of hard tick species. This parasitic wasp oviposits to feeding and unfed larval and nymphal tick hosts, but the development of the eggs only occurs in fully engorged nymphs [10]. Once hatched, larvae of the wasp begin to feed on the tick tissue, eventually causing its death. Unlike other parasitic Chalcidoidea, larvae of *I*. *hookeri* consume also the vertebrate blood ingested by the nymphs. This diet is unique among the parasitic species of Hymenoptera families [11]. Wasps emerge from their hosts through a single hole gnawed at the posterior end of the abdomen, where the cuticle is the thinnest [12].

Rickettsiae are well-known tick-associated, obligate intracellular Alfaproteobacteria [13]. Ecological characteristics of the tick vectors of rickettsiae influence the epidemiology and clinical aspects of tick-borne diseases [14]. Ticks may acquire rickettsiae through transovarial transmission (the transfer of bacteria from the adult female ticks to the subsequent generation of ticks via the eggs) and/or transstadial transmission, i.e. the transfer of bacteria from stage to stage [15].

In this study we developed a specific Real-Time PCR tailored for the detection of the *I*. *hookeri* DNA and aimed to find (1) an association between the presence of *A*. *nasoniae* and *I*. *hookeri* in ticks and (2) an influence of the parasitoids on rickettsial infection in ticks. Finally, we hypothesized how the bacteria and parasitoids could act in the developmental cycle of ticks to which the ontogenesis of wasps is connected.

## Material and Methods

### Tick collection

Host-seeking nymphal *I*. *ricinus* were collected by blanket dragging from vegetation in the campus of the Slovak Academy of Sciences (SAS) in Bratislava, 48.17°N, 17.07°E, altitude about 210 m, in April-May 2014 and at the beginning of September 2014. The SAS campus is a fenced area of 32 ha located on the south-western foothills of the Small Carpathians. It is characterized by patches of the original oak-hornbeam forest with admixture of beech, ash, black locust, maple, lime tree, elm, alder, and common hazel which are fragmented by roads, pavements, and built-up areas. No specific permissions were required for questing tick collections in this location as the SAS campus is not a protected area. The field study did not involve any endangered nor protected species.

### Parasitoid wasp eclosion

Nymphs collected in April-May 2014 were subsequently fed on laboratory Balb/c mice. Thirty *I*. *ricinus* nymphs were placed in a retaining chamber and glued on the shaved back of one mouse. Engorged nymphs were collected to 15 ml tubes closed with nylon mesh and perforated lid. Tubes were incubated in a desiccator at room temperature and 85% relative humidity. Engorged nymphs were evaluated for the presence of parasitoid wasps, and/or moulting to adult ticks. The time period from the detachment of engorged nymphs to the emergence of parasitoid wasps ranged from 20 to 30 days. The parasitoids that emerged were separated into test tubes and deep-frozen at -80°C until subsequent analyses. The sex ratio (males to females) of adult wasps was evaluated by microscopic examination.

### Extraction of DNA

DNA was extracted from emerged *I*. *hookeri* wasps and host-seeking *I*. *ricinus* nymphs collected in September 2014, using an EZ1 Advanced XL automated extractor (Qiagen), and Qiagen manufacturer’s kit, according to supplier’s instructions. DNA was stored at 4°C until further use.

### Polymerase chain reaction

Based on cytochrome oxidase subunit I sequences of *I*. *hookeri* available in GenBank (JQ315225.1), we designed primers and a Taqman probe for Real-Time PCR to specifically detect *I*. *hookeri*: Iphag583f 5′-TTGCTGTTCCAACAGGAGTAAA-3′ and Iphag820r 5′-CAAAAAATTGCAAAAACTGC-3′ and probe Iphag612s 6FAM^®^-AGATGATAAGCTTCAATAAATGGAA-TAMRA^®^. DNA extracted from *I*. *hookeri* (obtained from parasitized *I*. *ricinus* nymphs in 2013) served as a positive control when using the primers and probe targeting parasitoid DNA in ticks. The set of primers/probe was verified for specificity with 30 negative controls (DNAs extracted from 20 bacterial, 5 arthropod and 5 vertebrate species).

PCRs were carried out in a CFX 96 Real-Time system C 1000 Thermal Cycler controlled by the vendor software (BioRad). The 20 μl of PCR mixture included 10 μl of the Takyon No ROX Probe 2x MasterMix UNG (Eurogentec), 0.5 μl (20 pmol.μl^-1^) of each primer, 0.5 μl (20 pmol.μl^-1^) of probe, 3.5 μl of milliQ water and 5 μl of extracted DNA. The amplification conditions were as follows: an initial denaturation step at 95°C for 3 min, followed by 40 cycles of denaturation at 95°C, annealing and elongation at 60°C for 60 s, with fluorescence acquisition in single mode. To avoid false-negative results, each sample was run in technical triplicate. A mean cycle threshold (*C*_t_) value below 35 indicated the sample as positive, and a *C*_t_ value above 35 indicated the sample as positive only if another two sets were positive [16].

*Arsenophonus nasoniae* was detected by *rpoB* gene-based Real-Time PCR [17] in emerged parasitoid wasps and unfed *I*. *ricinus* nymphs. As a positive control we used DNA extracted from *A*. *nasoniae* cultured on Columbia agar [17].

To screen wasps and nymphs for the presence of all spotted fever group rickettsiae (SFG), we used a previously published PCR assay with *Rickettsia*-specific *gltA* gene–based RKND03 system [18]. If the first screening was positive, a second directed step of molecular screening was performed to target rickettsiae at the species level using various sets of primers and probes [16;19]. DNA extracted from the cell culture supernatant of *Rickettsia montanensis* served as a positive control when using the primer and probe set targeting SFG *Rickettsia*; DNA extracted from the cell-culture supernatant of each particular *Rickettsia* species served as a positive control for the corresponding primer and probe set.

### Ethics statement

The usage of animals in the experiment was approved by the State Veterinary and Food Administration of the Slovak Republic (permit number 1335/12-221). The mice were euthanized at the end of the study by cervical dislocation. The experiments were performed under standard conditions in the experimental animal facility of the Institute of Virology, Slovak Academy of Sciences (Permit number: SK P 01014). No specific permissions were required for locations where collecting of questing ticks was carried out. The field studies did not involve any endangered nor protected species.

## Results

We screened for bacterial positivity *I*. *hookeri* adults that emerged from engorged *I*. *ricinus* nymphs, collected in questing stage from vegetation in April-May 2014 and subsequently fed on laboratory mice, and in unfed host-seeking nymphs collected in September 2014.

### Engorged tick nymphs and emerged *I*. *hookeri* wasps

**Fig 1:**
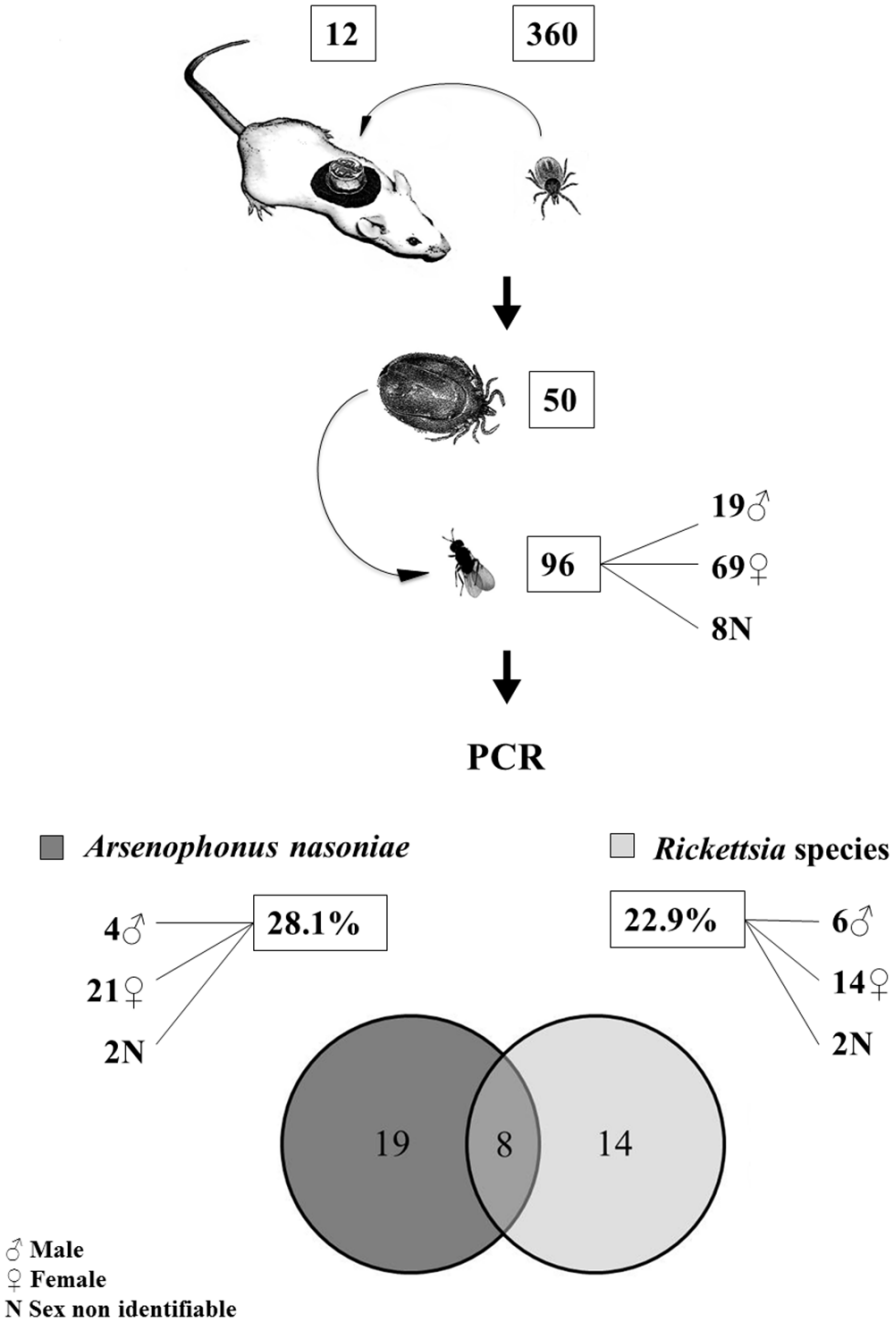
Workflow of laboratory experiments. A total of 360 *I*. *ricinus* nymphs were fed on 12 Balb/c mice. Of those, 50 engorged nymphs were parasitized by *I*. *hookeri* wasps. The obtained 96 parasitoids were subsequently screened for the presence of *A*. *nasoniae* and rickettsiae by PCR. The DNA of *A*. *nasoniae* was found in 27 wasps (28.1%), *Rickettsia* sp. in 22 wasps (22.9%). Eight wasps were positive for both bacteria—*A*. *nasoniae* and *Rickettsia* sp.

A total of 360 *I*. *ricinus* nymphs (Fig 1), used for infestation of laboratory mice, were engorged and detached. Of those 50 nymphs (13.8%) were parasitized by *I*. *hookeri*. Numbers of wasps emerging from each individual nymph were not exactly evaluated but, they were ranging from 2–20. We collected the adult wasps soon after emerging and stored at -80°C for further purposes.

**Fig 2:**
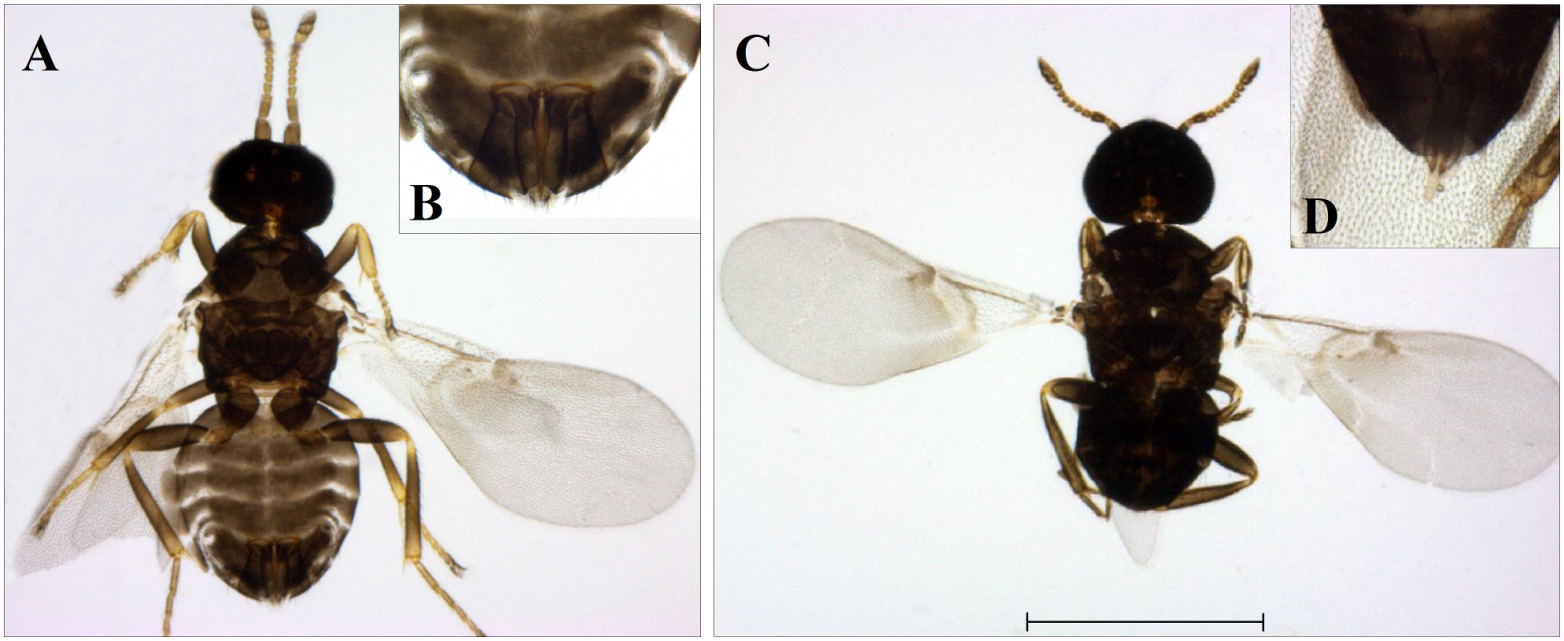
*Ixodiphagus hookeri* adults. (A) Habitus of a female after treatment with *Marc André* solution. (B) Magnified female genitalia. (C) Habitus of a male. (D) Magnified male genitalia. Scale bar represents 0.5 mm.

Determination of the sex of the wasps was done by microscopic examination. Emerged females were mainly black coloured and their length was around 1 mm. Males resembled females beside differences in genitalia and antennae (Fig 2). The sex ratio (males to females) of adult wasps was 1:3.6. We identified 19 males, 59 females, but were not able to assign the sex of 8 wasps (Fig 1), which was likely due to improper handling.

### *Ixodiphagus hookeri* harbours the endosymbiotic bacterium *Arsenophonus nasoniae*, and *Rickettsia* sp.

All *I*. *hookeri* obtained from parasitized nymphs were screened for the presence of *A*. *nasoniae* using specific primers for its *rpoB* gene. DNA of this bacterium was detected by Real-Time PCR, in 28.1% of the parasitoids, mainly females. Surprisingly, *A*. *nasoniae* was also identified in four male wasps, despite of its well-known reproductive parasitism causing male offspring mortality.

Furthermore, all 96 wasps were tested for the presence of *Rickettsia* species. We found that 22.9% of examined wasps contained DNA of rickettsiae (Fig 1). After amplification with specific primers, we detected *Rickettsia helvetica* in 13.5% (4 males, 8 females, 1 sex non identifiable) and *Rickettsia monacensis* in 9.4% (2 males, 6 females, 1 sex non identifiable) of wasps. Interestingly, eight parasitoids were positive for the presence of both *A*. *nasoniae* and *Rickettsia* sp. (*R*. *helvetica* or *R*. *monacensis*).

### Bacterial infection rates in the natural population of *I*. *ricinus* nymphs due to presence of *I*. *hookeri*

**Fig 3:**
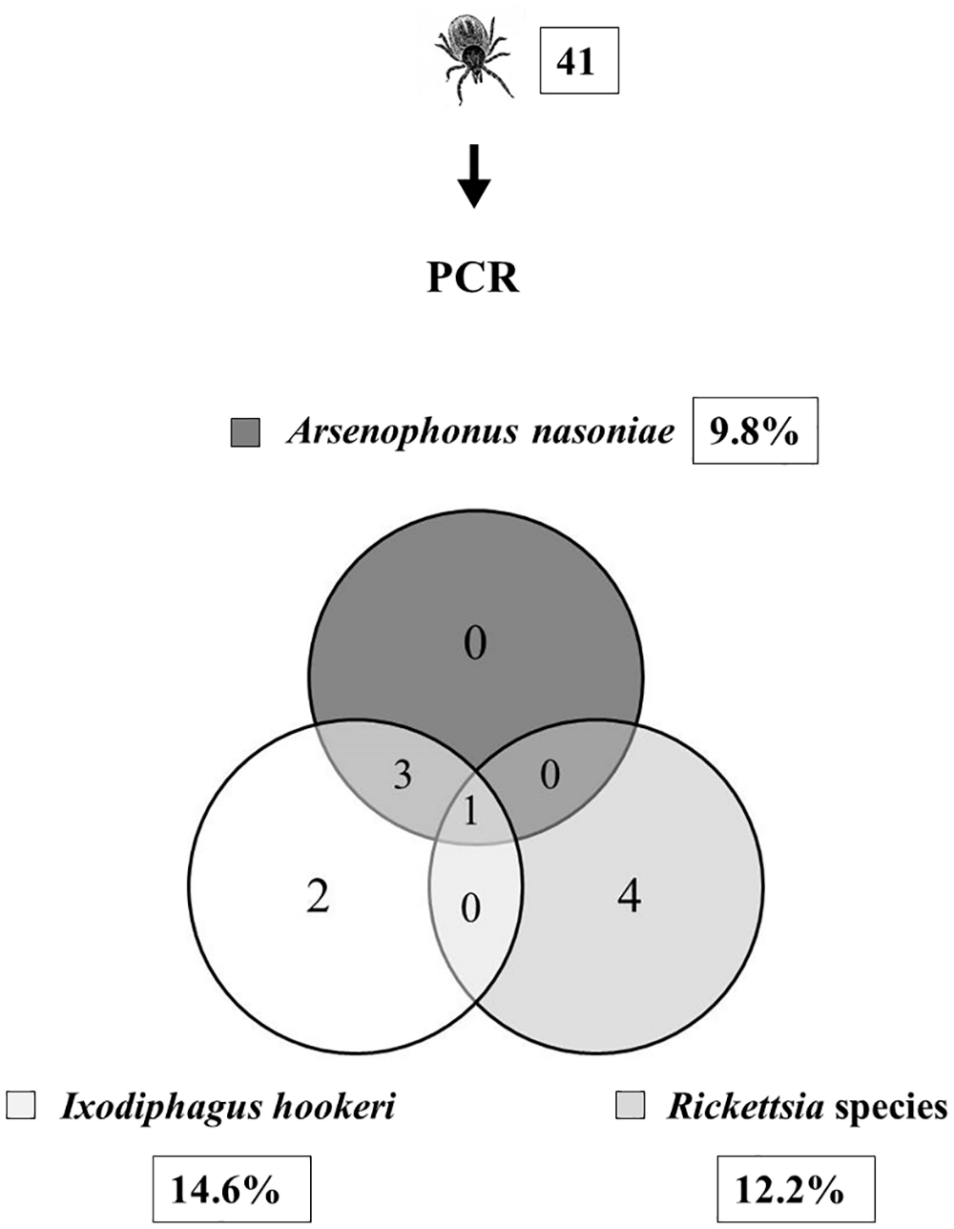
Detection of bacteria and wasps in unfed *I*. *ricinus* nymphs. Overall 41 host-seeking *I*. *ricinus* nymphs were examined for the presence of *A*. *nasoniae* and *Rickettsia* sp. as well as for *I*. *hookeri* by PCR. The DNA of *A*. *nasoniae* was successfully amplified in 4 nymphs (9.8%) and the presence of rickettsiae was confirmed in 5 nymphs (12.2%). Six nymphs were parasitized by *I*. *hookeri* wasps (14.6%). As shown in the Venn-diagram, all nymphs that were positive for *A*. *nasoniae* were simultaneously parasitized by *I*. *hookeri*. Co-infection by both bacteria occurred only in one *I*. *ricinus* nymph which also contained wasp DNA.

DNA was extracted from 41 host-seeking *I*. *ricinus* nymphs, collected from vegetation in September 2014 (Fig 3). They were directly tested for the presence of the bacteria, *A*. *nasoniae* and/or *Rickettsia* sp., as well as for the presence of *I*. *hookeri* DNA by Real-Time PCR. Out of all investigated host-seeking nymphs, 14.6% contained wasp’s DNA, which was comparable to the value, obtained for engorged nymphs (13.8%) that originated from the same collection site. Strikingly, we identified *A*. *nasoniae* in a lower percentage of questing nymphs (9.8%) than in the emerged parasitic wasps (28.1%).

All nymphs that were positive for *A*. *nasoniae* also hosted *I*. *hookeri*, suggesting that the presence of the bacterium depends not on the developmental stage of the tick but on the parasitisation of *I*. *ricinus* by the wasp. Two nymphs that were positive for *I*. *hookeri* were negative for *A*. *nasoniae*. Summing up, not all wasps harboured *A*. *nasoniae* but this bacterium was detected exclusively in those *I*. *ricinus* nymphs that carried *I*. *hookeri*. Thus, we may hypothesise, that the presence of *I*. *hookeri* in *I*. *ricinus* nymphs is correlated with the presence of *Arsenophonus nasoniae*.

The prevalence of *I*. *ricinus* nymphs infected by rickettsiae was 12.2% (Fig 3). One of these nymphs was found to harbour both *A*. *nasoniae* and *Rickettsia* sp., and was simultaneously parasitized by *I*. *hookeri*.

## Discussion

In this study we focused our attention to three parasitic organisms of ticks: the wasp *I*. *hookeri*, and the bacteria *A*. *nasoniae* and *Rickettsia* sp.

Among arthropod vectors, ticks harbour the largest diversity of microorganisms, ranging from viruses (tick-borne encephalitis), to bacteria (*Rickettsia* sp., *Borrelia* spp., species from Anaplasmataceae family, etc.) and/or eukaryotes (*Babesia* sp., *Theileria* sp.) [20;21]. Ticks are also hosts of macroparasites; including wasps such as *I*. *hookeri* (Table 1), which carry their own microbiome [21]. Normally, many insect species are simultaneously infected by multiple microbial symbionts, which in turn interact with each other, an co-regulate the biological processes of the host [22].

**Table 1:** Reports on *Ixodiphagus hookeri* detected in hard ticks.

Tick Species	References
*Ixodes ricinus*	[21;23–28]
*Ixodes scapularis*	[10;29–32]
*Ixodes holocytus*	[33]
*Ixodes tasmani*	[33]
*Amblyomma variegatum*	[34–37]
*Rhipicephalus sanguineus*	[38–40]
*Haemaphysalis concinna*	[41]
*Haemaphysalis bancrofti*	[33]
*Haemaphysalis bremneri*	[33]

The parasitisation rate of the *I*. *ricinus* nymphs, fed on laboratory Balb/c mice, by *I*. *hookeri* was 13.8%. Similarly, we found that 14.6% of questing *I*. *ricinus* nymphs contained wasps DNA. This agrees with the parasitation rates reported by Hu *et al*. [42] and Stafford *et al*. [31], but is higher compared to the investigated occurrence in Germany [25] and Italy [23]. The parasitisation rate of engorged nymphs might be even higher, since ticks are more likely parasitized while feeding on their vertebrate hosts [26]; wasp females visually evaluate and choose feeding nymphs over questing nymphs, because a feeding nymph may be an immediately available source of a meal for parasitoid larvae [37].

We discovered that natural populations of the wasp *I*. *hookeri* are infected at a 28.1% prevalence by *A*. *nasoniae*. Remarkably, *A*. *nasoniae* has never been reported to be associated with *I*. *hookeri* wasps before. This bacterium is well-known for its unique evolutionary evolved male-killing phenomenon, eliminating the male offspring of the wasps [3;6]. This is achieved by inhibition of the production of maternal centrosomes, organelles required specifically for early male embryonic development—male arise from unfertilized, haploid eggs and obtain their centrosomes maternally [43]. In that context we also found that the sex ratio of emerging adult parasitoids was strongly female biased (19♂:69♀). The obtained data were similar to those reported by Davis *et al*. [12], or Collatz *et al*. [25]. Mostly female wasps (77.78%, 1:5.25 ratio of infected males to females, in two instances sex non identifiable) were positive for *A*. *nasoniae*. We were able to identify only four adult males to be positive for *A*. *nasoniae* using Real-Time PCR.

*Arsenophonus nasoniae* has been identified in seven hard tick species (Table 2), but thus far there has been no clear understanding of the nature of the bacterium-host relationship in ticks. Using primers specific to *I*. *hookeri* and *A*. *nasoniae*, we showed that in an *I*. *ricinus* population, all individual ticks harbouring *A*. *nasoniae* were parasitized by *I*. *hookeri*, while ticks without wasps were *Arsenophonus*-free. Keeping in mind, that the simple detection of a vector-borne bacterial agent in a parasite does not demonstrate vector competency [44], it seems that the presence of *A*. *nasoniae* may be linked to the presence of wasp parasitoids in those ticks.

**Table 2:** Published studies on detection of *Arsenophonus nasoniae* in ticks.

Tick Species	References
*Dermacentor variabilis*	[45–48]
*Dermacentor silvarum*	[49]
*Dermacentor andersoni*	[47;50]
*Ixodes scapularis*	[45;46]
*Ixodes ricinus*	[17;51]
*Haemaphysalis longicornis*	[52]
*Amblyomma americanum*	[46;47;53]

This assumption was already foreseen in our earlier study, in which the infection rate was comparable, namely, *A*. *nasoniae* was identified in 20 *I*. *ricinus* individuals; only one adult tick out of 28 and 19 nymphs out of 52 were positive when screened by specific PCR [51]. We proposed that the presence of *A*. *nasoniae* in ticks depends on their developmental stage. In the current study we hypothesise that *A*. *nasoniae* is present only due to *I*. *hookeri* parasitoids.

Several authors already proved [25;42;54] that larvae of the *I*. *hookeri* wasp consume the entire tissue contents of the engorged nymphal tick, including the blood meal ingested by the host nymph, which may be infected by a variety of microorganisms. In the present study we obtained important data about the presence of rickettsiae in emerged *I*. *hookeri* wasps. We detected *R*. *helvetica*, and/ or *R*. *monacensis* for the first time in adults of this parasitoid species. Almost 23% *Rickettsia*-positive individuals were confirmed by Real-Time PCR.

**Fig 4:**
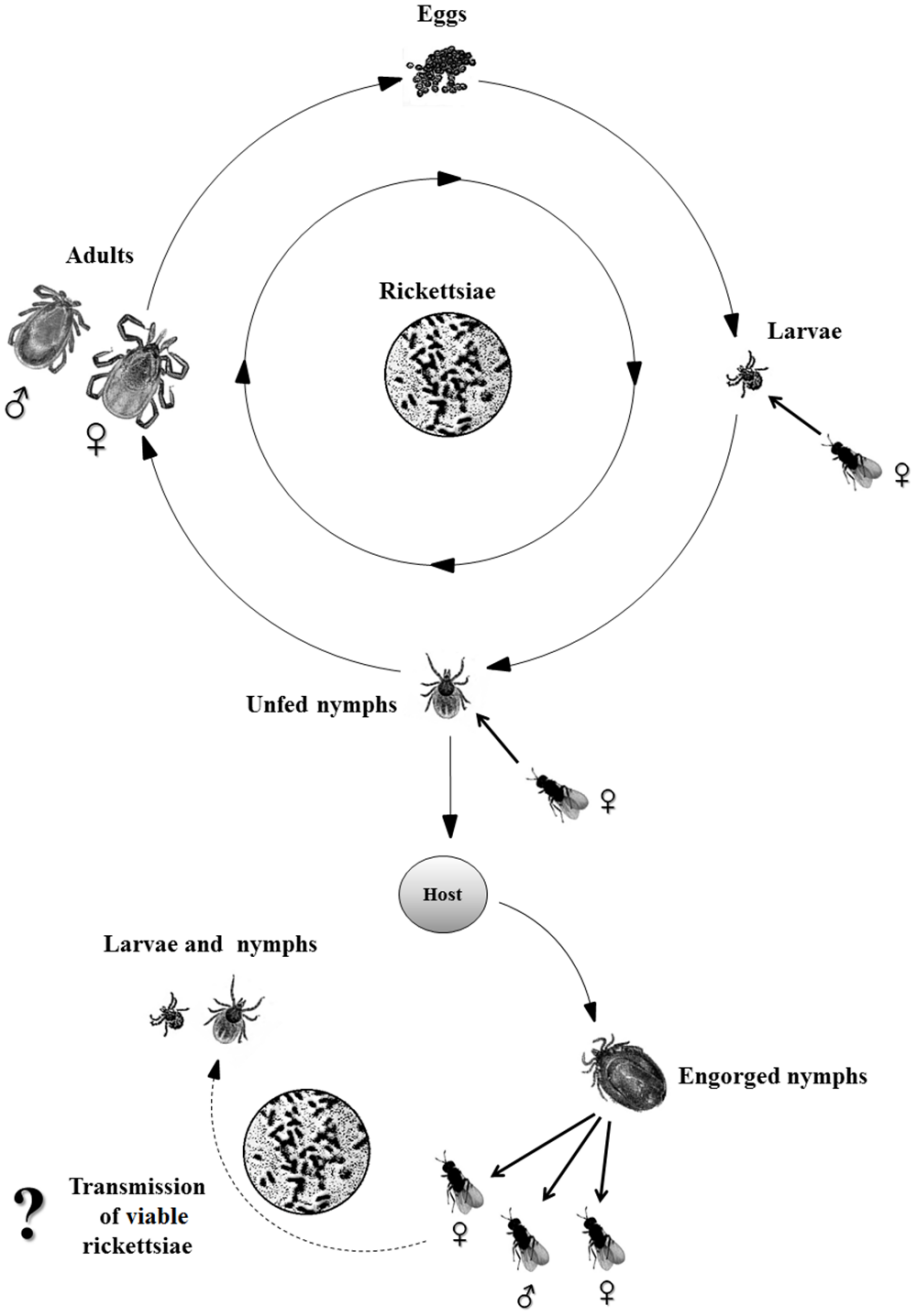
The questionable role of parasitic wasps in the transmission of rickettsiae, illustrated on the tick’s life cycle. Adult female *I*. *hookeri* oviposits in larvae and nymphs of ixodid ticks, but the wasp eggs start to develop only in fully engorged nymphs. The immature parasitoid wasps consume the nymph’s tissue and its ingested blood meal, causing nymph death. During the tick’s life cycle (eggs, larvae, nymphs, adults), rickettsiae can pass from stage to stage. In our experiments we successfully amplified rickettsial DNA not just in unfed nymphs but also in emerged adult wasps. More experiments will be needed to demonstrate if *I*. *hookeri* may act as a biological vector of *A*. *nasoniae* and *Rickettsia* sp.

However, we do not know whether the detected rickettsial DNA originated from consumed *Rickettsia*-infected tick cells or viable rickettsiae (Fig 4). Follow-up studies are needed to clarify the capability of *Ixodiphagus* wasps to carry viable rickettsiae, thus to transmit them to ticks transovarially.

## Conclusions

We detected *A*. *nasoniae* in adult *I*. *hookeri* wasps for the first time. The natural population of the *I*. *hookeri* wasp was infected by *A*. *nasoniae* at a 28.1% prevalence. The parasitisation rate of *I*. *ricinus* nymphs originating from the same natural site, fed or unfed, by *I*. *hookeri* was comparable (13.8% and 14.6%, respectively). Nymphs that were not parasitized by wasps were *Arsenophonus*-free. Unique in our study was also the molecular detection of rickettsial DNA in 23% of *I*. *hookeri* adults. However, the transmission of viable rickettsiae from tick to tick by a wasp deserves further investigation.
